# Feedforward discharges couple the singing central pattern generator and ventilation central pattern generator in the cricket abdominal central nervous system

**DOI:** 10.1007/s00359-019-01377-7

**Published:** 2019-11-05

**Authors:** Stefan Schöneich, Berthold Hedwig

**Affiliations:** 1 Department of Zoology, University of Cambridge, Cambridge, UK; 2 Institute of Zoology and Evolutionary Research, Friedrich-Schiller-University Jena, Jena, Germany

**Keywords:** Fictive singing, Abdominal ventilation, Central pattern generation, Corollary discharge, Motor systems coordination

## Abstract

We investigated the central nervous coordination between singing motor activity and abdominal ventilatory pumping in crickets. Fictive singing, with sensory feedback removed, was elicited by eserine-microinjection into the brain, and the motor activity underlying singing and abdominal ventilation was recorded with extracellular electrodes. During singing, expiratory abdominal muscle activity is tightly phase coupled to the chirping pattern. Occasional temporary desynchronization of the two motor patterns indicate discrete central pattern generator (CPG) networks that can operate independently. Intracellular recordings revealed a sub-threshold depolarization in phase with the ventilatory cycle in a singing-CPG interneuron, and in a ventilation-CPG interneuron an excitatory input in phase with each syllable of the chirps. Inhibitory synaptic inputs coupled to the syllables of the singing motor pattern were present in another ventilatory interneuron, which is not part of the ventilation-CPG. Our recordings suggest that the two centrally generated motor patterns are coordinated by reciprocal feedforward discharges from the singing-CPG to the ventilation-CPG and vice versa. Consequently, expiratory contraction of the abdomen usually occurs in phase with the chirps and ventilation accelerates during singing due to entrainment by the faster chirp cycle.

## Introduction

In all animals the timing of rhythmic muscle activity during simultaneous repetitive motor behaviors is well coordinated (von Holst 1935, 1943; Kutsch 1969; Syed and Winlow 1991; Dick et al. 1993; Chrachri and Neil 1993; Ramirez 1998; Boggs 2002; Moore et al. 2014; Stein 2018). The coupling between the motor cycles can range from strict synchrony in absolute coordination to periodic phase coupling as in relative coordination (von Holst 1935; Berger et al. 1970; Bramble and Carrier 1983; Kawahara et al. 1989; Paripovic et al. 1996; Moore et al. 2014; Hao and Berkowitz 2017). Repetitive motor activity is generally produced by central pattern generators (CPGs), networks of interneurons within the central nervous system that generate rhythmic activity, even in the absence of sensory feedback (Delcomyn 1980; Marder and Bucher 2001; Mulloney and Smarandache 2010; Selverston 2010). Coordinated motor patterns can be generated by the same multifunctional CPG network, partly overlapping CPG networks, or by entrainment between two distinct CPGs (von Holst 1936; Bartos et al. 1999; Bucher et al. 2006; Briggman and Kristan 2008; Rillich et al. 2013; Hao and Berkowitz 2017). When the activity cycles of two motor patterns are strictly phase-coupled in a one-to-one manner both patterns may be driven by the same CPG network. If the timing of the two motor patterns is at least temporarily uncoupled, distinct CPGs pace the two rhythms, which may become phase-coupled by a secondary mechanism. Such coupling between two CPG networks can be either mutual or hierarchically organized such that one rhythm entrains the other (von Holst 1935, 1943; Robertson and Moulins 1981) and central neural mechanisms of motor pattern coordination at the neuronal level are emerging (Dickinson 1995; Clarac and Pearlstein 2007; Selverston 2010). For example, in the stomatogastric system of crustaceans the rhythmic synaptic input from the pyloric CPG can modulate the activity of the gastric mill CPG (Nadim et al. 1998; Bartos et al. 1999; Nusbaum and Beenhakker 2002), in the crayfish abdominal swimmeret system gradients of synaptic strength underlie the coordination of swimmerets (Smarandache et al. 2009), and evidence points to local CPGs being weakly coupled to support inter-leg coordination in walking insects (Knebel et al. 2016; Bidaye et al. 2017; Daun et al. 2019).

Here we investigated the neural coordination between the motor patterns of abdominal ventilation and the generation of chirps in fictively singing field crickets (*Gryllus bimaculatus* de Geer). Male crickets sing by rhythmic opening and closing movements of their forewings, each closing movement generates a short sound syllables or pulse. In the calling song of *G. bimaculatus*, chirps with 3–5 syllables are repeated at a rate of about 3 Hz with a period of 30–40 ms (Kutsch 1969; Jacob and Hedwig 2016). Unlike in many vertebrates (Suthers et al. 1999; Ashmore et al. 2008; Andalman et al. 2011), for sound producing insects a strict coordination between sound production and breathing may not be a functional necessity. Nevertheless, a strict phase-coupling between chirping and the expiration cycles of abdominal ventilation occurs in singing crickets (Huber 1960; Kutsch 1969; Kutsch and Huber 1989; Otto and Hennig 1993; Paripovic et al. 1996). Kutsch (1969) suggested that a slow oscillator network may function as the common timer for the ventilatory rhythm and the calling song chirps, and a faster network would provide the basis for the syllable pattern and the flight motor pattern. In view of neuronal efficiency, it seems intriguing that overall fewer neurons may be needed if one network would provide for both, the activity underlying ventilation as well as the chirp pattern during sound production (see Kutsch 1969; Bentley 1969; Kutsch and Huber 1989). The rhythm generating network for ventilation in orthopteran insects is located in the metathoracic and subsequent abdominal ganglia (Huber 1960; Miller 1960, 1966; Lewis et al. 1973; Ramirez and Pearson 1989a; Burrows 1996; Bustami and Hustert 2000) which in crickets is the same part of the central nervous system that houses the singing-CPG for the calling song (Hennig and Otto 1995; Schöneich and Hedwig 2011, 2012, 2017; Jacob and Hedwig 2016, 2019). Thus, in crickets abdominal ventilation and singing are suited behaviors to unravel the neuronal basis of central motor pattern coordination.

## Materials and methods

### Animals

Male Mediterranean field crickets (*Gryllus bimaculatus* DeGeer) were selected 1–2 week after their final molt from the cricket colony maintained at about 27 °C on a 12 h:12 h light:dark cycle in the Department of Zoology (University of Cambridge, UK). Experiments were carried out at room temperature (22–26 °C).

### Preparation and brain injection

After removing legs and wings, the crickets were opened by a dorsal longitudinal incision and pinned out ventral side down onto a plasticine covered platform. The ganglia of the ventral nerve cord were exposed for electrophysiological recording and continually perfused with Ringer’s saline (concentrations in mmol l^−1^: NaCl 140, KCl 10, CaCl_2_ 7, NaHCO_3_ 8, MgCl_2_ 1, *N*-trismethyl-2-aminoethanesulfonic acid 5, d-trehalose dihydrate 4; adjusted to pH 7.4). The head of the cricket was waxed to a moveable metal support, and a small window was cut in the forehead cuticle to gain access to the brain. The peripheral nerves of all three thoracic (T1, T2, T3-A2) and the first unfused abdominal ganglion (A3) were cut. Fictive singing was elicited by pressure-injection (Pneumatic PicoPump PV820; WPI, Sarasota, FL, USA) of the acetylcholine esterase inhibitor eserine (10^−2^ M in saline; Sigma-Aldrich, St Louis, MO, USA) into the ventral protocerebrum using a blunt glass-microcapillary (Wenzel and Hedwig 1999; Schöneich and Hedwig 2012, 2015).

### Electrophysiological recordings

**Fig. 1:**
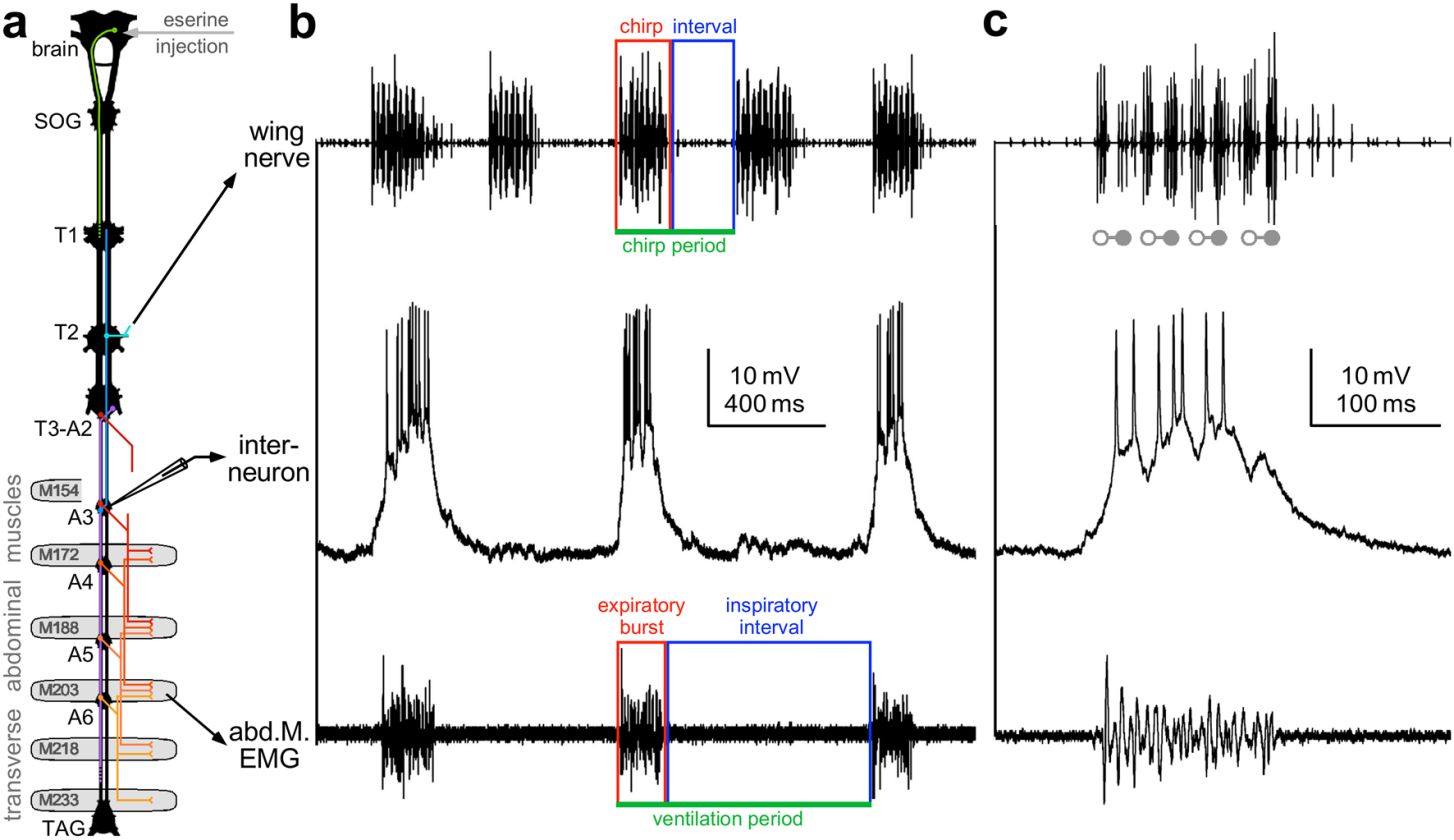
Experimental design. **a** Diagram of cricket nervous system indicating the eserine injection into the brain, the mesothoracic wing-nerve (T2-N3A) recording, intracellular recording in the abdominal ganglion (A3), and electromyogram (EMG) of an abdominal muscle (M203) supporting expiration. Orange symbols indicate motoneuron innervation pattern of transverse abdominal muscles, key interneurons of the singing network indicated in blue and the command neuron for calling song in green. **b**, **c** Recordings show the fictive singing motor pattern in the wing nerve (top trace), activity of an abdominal ventilatory interneuron (middle trace) and expiratory EMG activity (bottom trace). **b** Colored boxes highlight chirp duration (red), chirp interval (blue) and chirp period (green) of the singing motor pattern (top); and expiratory burst duration (red), inspiratory interval (blue) and ventilation period (green) in the EMG recording (bottom). Color code is different to code for neurons in CNS diagram. **c** Detailed view of wing nerve activity showing one four-syllable chirp with the activity of wing-opener and wing-closer motoneurons marked by *open* and *closed* symbols, respectively

Extracellular recordings were amplified with a differential AC-amplifier (Model 1700; A-M Systems, Sequim, WA, USA). The motor activity of fictive singing was recorded with a double-hook electrode from the mesothoracic nerve 3A (Fig. 1), we refer to this nerve branch as *wing nerve*, as it contains axons of wing-opener and wing-closer motoneurons. The generation of a fictive “syllable” is indicated by subsequent opener-closer activity. Electromyogram of the abdominal ventilatory activity was recorded by inserting two thin stainless-steel wires (30 µm diameter) that were varnish-coated but for the tips, in the transversal muscle of the 5th abdominal segment (TM5: Consoulas et al. 1993; M203: Kawasaki and Kita 1995). Transverse abdominal muscles support expiration by driving the dorso-ventral compression of the abdomen during ventilatory pumping. Contraction of these muscles is controlled both, within each segment by a bilateral pair of excitatory motoneurons in the corresponding abdominal ganglion supplying the left and right transverse abdominal muscles independently, and intersegmentally by peripheral collaterals of homologous motoneurons from adjacent segments (Consoulas et al. 1993; Kawasaki and Kita 1995).

For intracellular recording, the abdominal ganglion A3 was stabilized between a silver ring and a subjacent silver platform with an embedded optic fiber for brightfield illumination. Sharp microelectrodes were pulled (DMZ-Universal Puller, Zeitz-Instruments, Martinsried, Germany) from thick-wall borosilicate glass capillaries (GC100F-10, Harvard Apparatus Ltd., Kent, UK), filled with 2 mol l^−1^ potassium acetate and resistances of 70–90 MΩ. Intracellularly recordings were amplified using a DC-amplifier with current injection facility (BA-01X, NPI, Tamm, Germany).

### Data sampling and analysis

All electrophysiological recordings were monitored with an oscilloscope (Tektronix 5440) and digitized with 40 kHz sampling rate per channel (Micro1401 mk II; CED; Cambridge; UK) for storage on a PC hard drive. Off-line data analysis was performed with Spike2 (CED; Cambridge UK) and custom-made software Neurolab (Knepper and Hedwig 1997).

We categorized the neurons as ventilatory or singing interneurons if their membrane potential and spike activity was coupled to the respective motor rhythm. If spike activity preceded the motor activity and manipulation of activity by intracellular current injection altered the ongoing motor pattern, like resetting the rhythm by shifting the phase of the next cycle or changing the frequency of the rhythm by eliciting additional activity, we considered the neuron as part of the CPG network (cf. Schöneich and Hedwig 2012).

For quantitative analysis we either used the singing motor activity recorded from the wing nerve or the ventilatory muscle activity as reference. Time = 0 ms in the figures corresponds either to the first wing-opener spike of a fictive chirp in the wing nerve or the beginning of the ventilatory burst in the abdominal muscle, respectively. Before averaging, the extracellular recorded spikes were full-wave rectified to prevent cancelation of biphasic signal components.

Mean values are given with standard deviations (mean ± SD) for all normally distributed data. When data failed testing for Gaussian distribution (D’Agostino and Pearson omnibus normality test) the value of median, 5th–95th percentile, and the interquartile range (IQR) are given, statistical differences between datasets were tested with Mann–Whitney *U*-test and equality of variances was tested with *F*-test (Prism 5.0, GraphPad, La Jolla, CA, USA). In pooled data sets, each contributing animal is equally represented (*N* number of animals, *n* number of analyzed events; i.e., if the sampling size was *n* = 300 there were 30 data points from each of the *N* = 10 animals).

## Results

We analyzed the coordination between ventilation and singing and monitored the EMG of an abdominal expiratory muscle. We elicited fictive singing by microinjecting eserine in the brain (Fig. 1) and monitored singing by extracellular recording the wing-nerve activity. Intracellular recordings were obtained in abdominal ganglion A3, which houses interneurons of the singing-CPG (Schöneich and Hedwig 2012, 2017; Jacob and Hedwig 2019) and the ventilation-CPG (Ramirez and Pearson 1989a; Hustert and Mashaly 2013).

### Ventilatory motor pattern

**Fig. 2:**
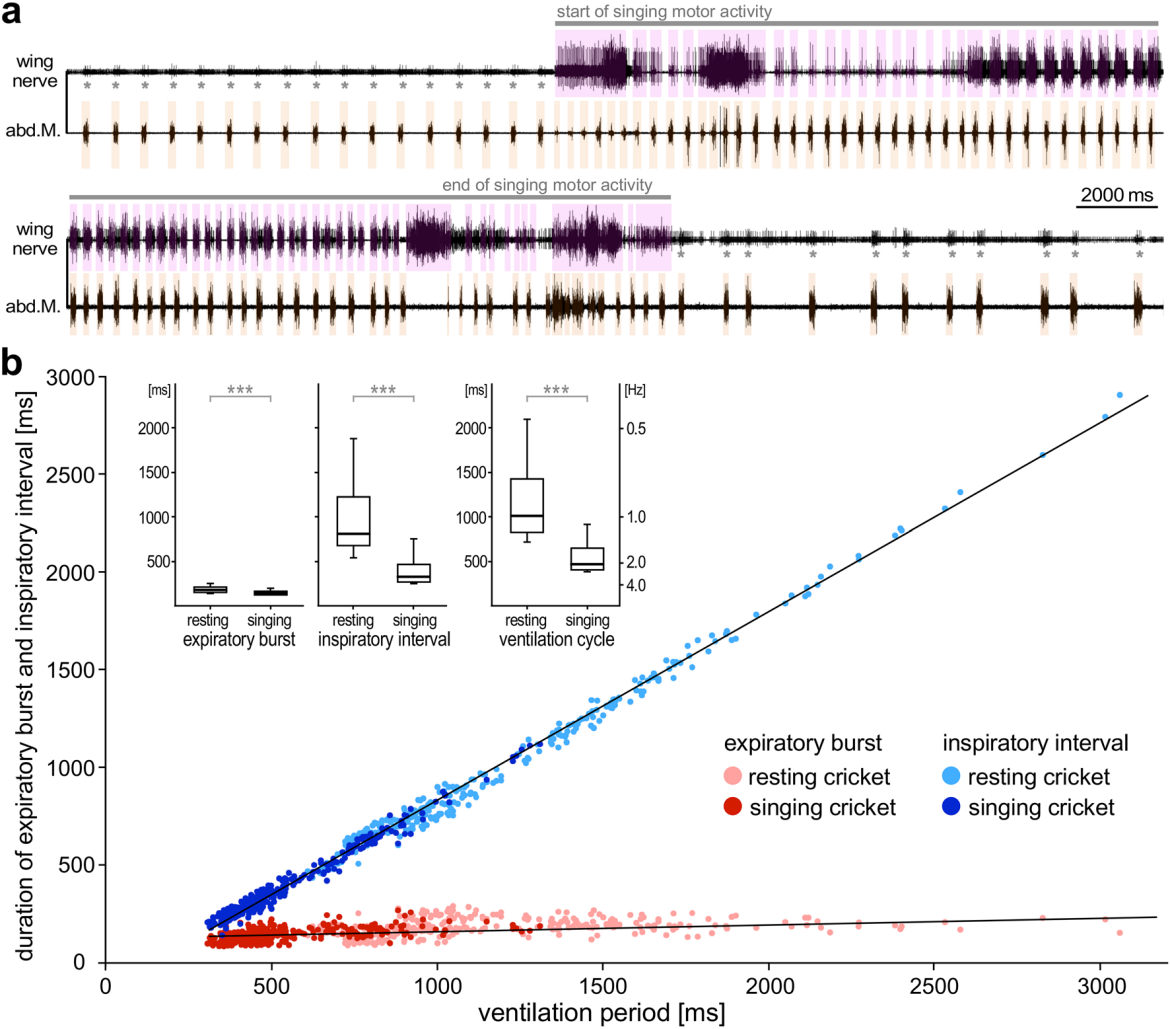
Singing accelerates ventilation by shortening the inspiratory interval. **a** The repetition rate of rhythmic abdominal muscle activity driving expiration (bottom trace, orange boxes) is increased during fictive singing monitored by wing nerve recording (top trace, magenta boxes). Asterisks mark groups of small spikes in the nerve recording that are coupled with expiration before and after singing. **b** Singing shortens the ventilation period by reducing the inspiration interval rather than the duration of expiration motor burst. Ventilation period and inspiration interval correlate strongly (linear fit: *R*^2^ > 0.99) while the duration of expiration bursts appears independent of the ventilation period (linear fit: *R*^2^ < 0.11). Same regression lines fit data before and during singing. Inset diagrams: box-and-whisker plots show median, IQR, 5 and 95 percentiles of the expiratory burst duration, inspiraton intervals and ventilation periods for crickets at rest and during singing (***Mann–Whitney *U*-test *P* < 0.0001). Data set from 10 animals: *n*_resting_ = 300, *n*_singing_ = 300)

Crickets power their gas exchange by rhythmic abdominal pumping movements, with phases of active expiration and passive inspiration. The abdominal compression is driven by expiration muscles, which subsequently relax during the passive inspiration phase (Huber 1960; Kawasaki and Kita 1995; Paripovic et al. 1996). Based on EMG recordings of the expiratory muscle M203 in *N* = 10 animals, the ventilatory motor pattern was quantitatively analyzed for *n* = 300 ventilatory cycles during singing and *n* = 300 ventilatory cycles in the resting animals (*n* = 30 for each condition in each animal). An EMG activity burst reflected the expiration phase in each ventilatory cycle, which in resting crickets was also indicated by a group of small spikes in the wing nerve recording (asterisks in Fig. 2a). During fictive singing these small spikes were obscured by the alternating bursts of the much larger wing-opener and wing-closer motoneuron spikes.

The ventilation periods were varied in the resting crickets over a wide range (IQR 828–1429 ms; *n* = 300). Within individual recordings, times of relatively uniform ventilation cycles took turns with times of rather irregular ventilation where expiratory bursts of muscle activity were followed by extended inspiratory intervals. A wide range of ventilation periods had also been reported in freely moving crickets (Koch 1981) and in locusts similar shifts between continuous and discontinuous ventilation pattern can be observed in the isolated nerve cord as well as intact animals (Bustami and Hustert 2000).

Our data show that the ventilation period was directly and linearly dependent on the inspiratory interval (Fig. 2b; linear fit: *R*^2^ > 0.99). The duration of expiratory motor bursts was rather constant (IQR 132–187 ms; *n* = 600) and independent of the ventilation period (Fig. 2b; linear fit: *R*^2^ < 0.11). Consequently, the variability of the ventilation period (inset diagrams in Fig. 2b) reflects the variability of the inspiratory interval (*F*-test: *F* = 1, *P* > 0.25; *n* = 600 each) rather than the variability of the expiratory burst (*F*-test: *F* = 118, *P* < 0.0001; *n* = 600 each).

The frequency of ventilation cycles was significantly elevated (Mann–Whitney *U*-test: *P* < 0.0001; *n* = 300 each; inset diagrams Fig. 2b) during fictive singing (median/IQR: 2.1 Hz/1.5–2.5 Hz) compared to the resting state (median/IQR: 1.0 Hz/0.7–1.2 Hz). The ventilation period during singing was also linearly dependent of the inspiratory interval and independent of the duration of the expiratory burst; and the same regression lines fit the data points for resting and singing condition (Fig. 2b). The expiratory bursts were about 20% shorter during singing than resting (median: 147 ms vs. 181 ms; Mann–Whitney *U*-test: *P* < 0.0001; *n* = 300 each). However, the more than 50% reduction of the ventilation period during singing (median: 470 ms vs. 1010 ms; Mann–Whitney *U*-test: *P* < 0.0001; *n* = 300 each) primarily reflects the significantly shorter inspiratory intervals (median: 329 ms vs. 812 ms; Mann–Whitney *U*-test: *P* < 0.0001; *n* = 300 each).

### Singing motor pattern

Injecting eserine into the frontal brain elicited fictive singing in most of the preparations within 2–20 min. Some of the crickets sang enduringly for 1–2 h, in others singing was repeatedly intermitted by long pauses. We quantitatively analyzed *n* = 1000 chirp cycles from *N* = 10 animals that had continuously sung for several minutes (100 chirps from each animal). A large majority of fictive chirps had 3–5 syllables (Fig. 1c) resembling the natural calling song (Jacob and Hedwig 2016). We also recorded fictive chirps with 6–10 syllables; a pattern that is typical for the rivalry song (Kutsch 1969; Otto 1971; Adamo and Hoy 1995). Once singing was in full swing there was often a smooth transition between patterns of fictive calling song and rivalry song and in some animals normal “calling” and long “rivalry” chirps alternated randomly.

**Fig. 3:**
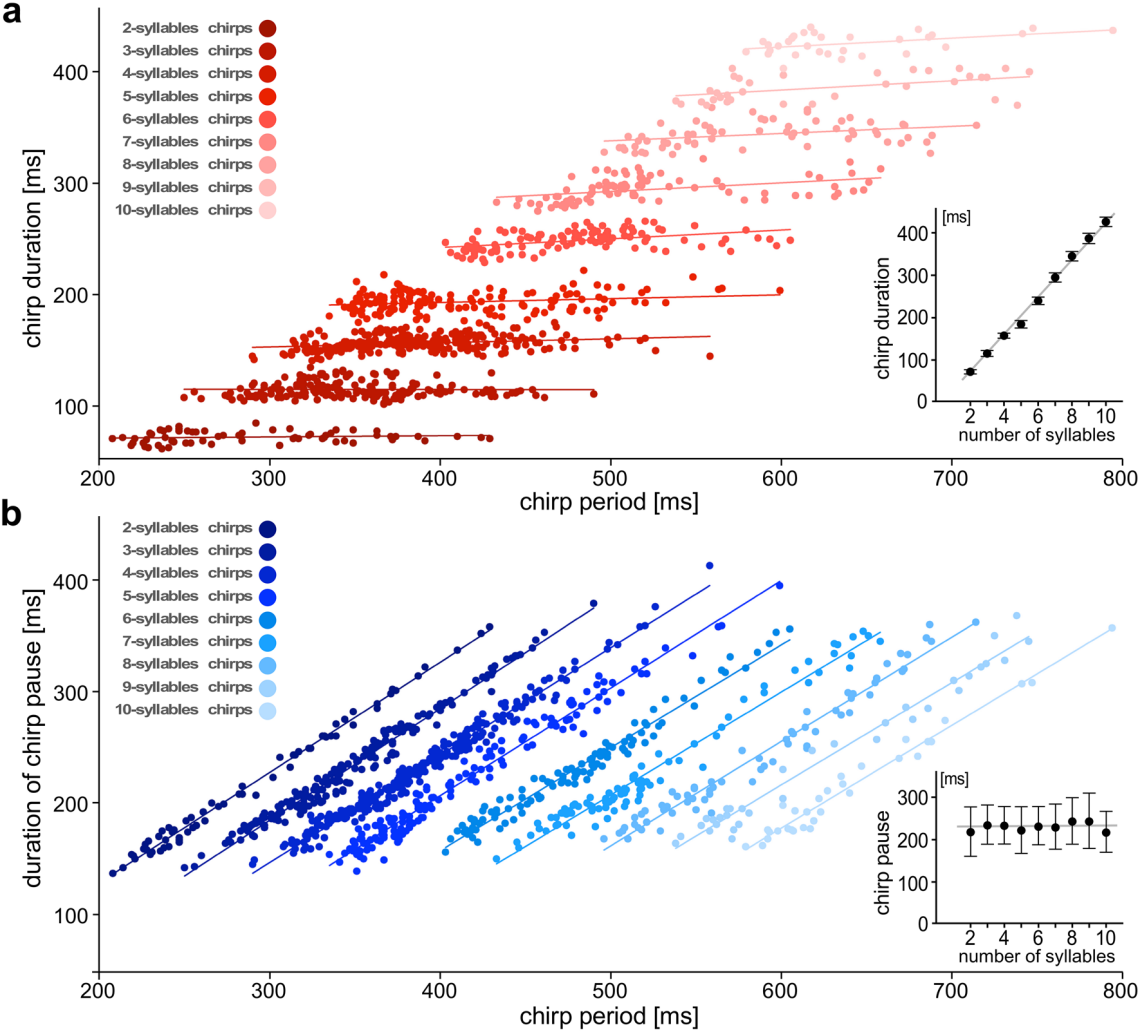
The chirp period is determined by the chirp interval and the number of syllables within a chirp. **a** For chirps with the same number of syllables/pulses the chirp duration is independent of chirp period (linear fits: *R*^2^ < 0.25 for each group). Inset diagram: The average chirp duration progressively increased by 39 ± 4 ms for each additional syllable in the chirp (*R*^2^ > 0.99 for linear fit). **b** For chirps with the same number of syllables there was a strong correlation between chirp interval and chirp period (linear fits: *R*^2^ > 0.97 for each group). Inset diagram: The chirp interval was independent of the number of syllables per chirp (*R*^2^ < 0.05 for linear fit). Data set from 10 animals: *n* = 1000 chirps

With increasing number of syllables, the mean syllable repetition rate of the chirps was gradually decreasing from 28 Hz for 2-syllable chirps to 24 Hz for 10-syllable chirps (mean ± SD for all chirps: 26 ± 1 Hz; *n* = 1000). Chirp duration closely correlated with the number of syllables in a chirp (inset diagram Fig. 3a; linear fit: *R*^2^ > 0.99), whereas chirp intervals were independent of syllable number and chirp duration (inset diagram Fig. 3b; linear fit: *R*^2^ < 0.05). The average chirp interval was 220 ms (median; IQR 190–260 ms; *n* = 1000); and fitting the linear equation “number of syllables multiplied by 39 ± 4 ms” the average chirp duration ranged from 73 ± 5 ms for 2-syllable chirps up to 420 ± 12 ms for 10-syllable chirps (mean ± SD). Similar to the ventilatory motor rhythm, the chirp period also directly correlated with the inter-chirp interval for chirps with the same number of syllables (Fig. 3b, linear fits: *R*^2^ > 0.97 each) but was independent of chirp duration (Fig. 3a, linear fits: *R*^2^ < 0.25 each).

### Motor pattern coupling

**Fig. 4:**
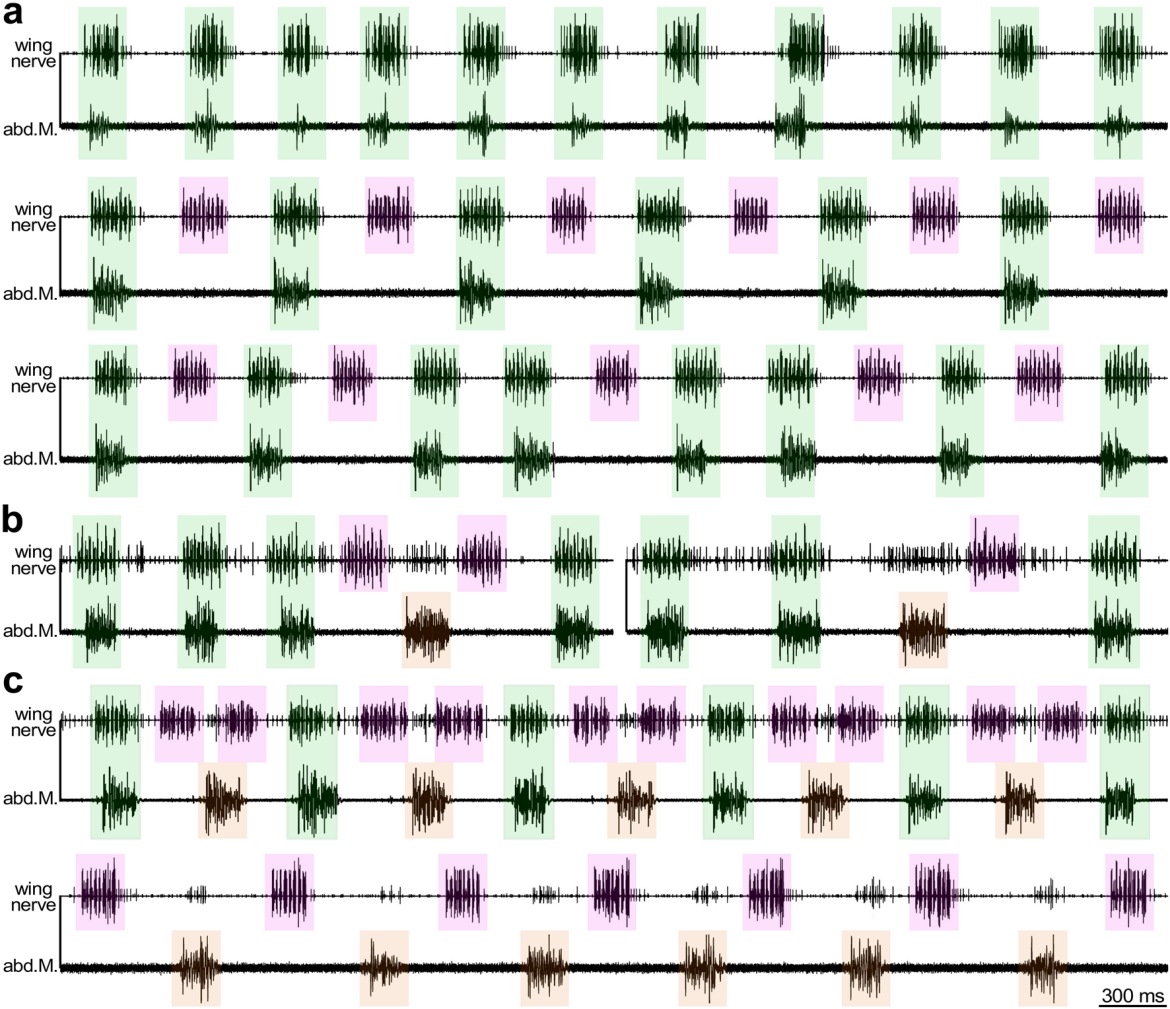
Coupling between singing and ventilation. **a**–**c** Recordings of the singing motor pattern (wing nerve) and expiratory activity (abdominal muscle). **a** During fictive singing either each or every second chirp is accompanied by expiratory activity. **b** Occasionally the coupling between chirps and expiration fails, the two motor rhythms resynchronized within the subsequent ventilation cycle. **c** Antiphase alternation between chirps and expiration occurred only in rare cases when the chirp frequency was either exceptionally high or low. Green background: synchronous chirps and expiration; magenta: chirps by their own, orange: expiration by its own

The ventilatory abdominal pumping became faster during fictive singing and synchronized to different degree with the chirp pattern. We generally observed either a strict 1:1 or 2:1 coupling between chirps and expiratory muscle activity (Fig. 4a top, middle). During the 1:1 coupling chirp activity and ventilatory activity occurred at the same time, while during the 2:1 coupling every other chirp was not accompanied by abdominal muscle activity; also obvious in the recordings of Kutsch (1969, Fig. 27). Occasionally we observed periods of continuing alternation between the 1:1 and 2:1 coupling (Fig. 4a, bottom). In all these cases expiratory EMG bursts were coupled to the generation of chirps. In some animals however, coupling between singing and ventilation could be more complex and transiently the ventilatory bursts were not coupled to a chirp. The expiration phase was then accompanied by a sequence of “small” spikes in the wing nerve (Fig. 4b), which may represent a subset of ventilation coupled motor units.

Occasionally unstable coupling between both motor patterns occurred. We observed a 3:2 coupling, during which a ventilatory burst was coupled to a chirp, while the next ventilation burst occurred right in the interval of the subsequent two chirps (Fig. 4c, top). Also a strict alternation between chirp and ventilation motor bursts occurred at very rare occasion, during which the chirp motor activity was not mirrored in the activity of the ventilation muscle, while the ventilatory pattern was reflected by groups of small-amplitude spikes in the wing nerve recording (Fig. 4c, bottom). Such periods of continuing unstable coupling or anti-phasic alternation between chirping and expiratory burst occurred only when the chirp frequency was either exceptionally high or low.

**Fig. 5:**
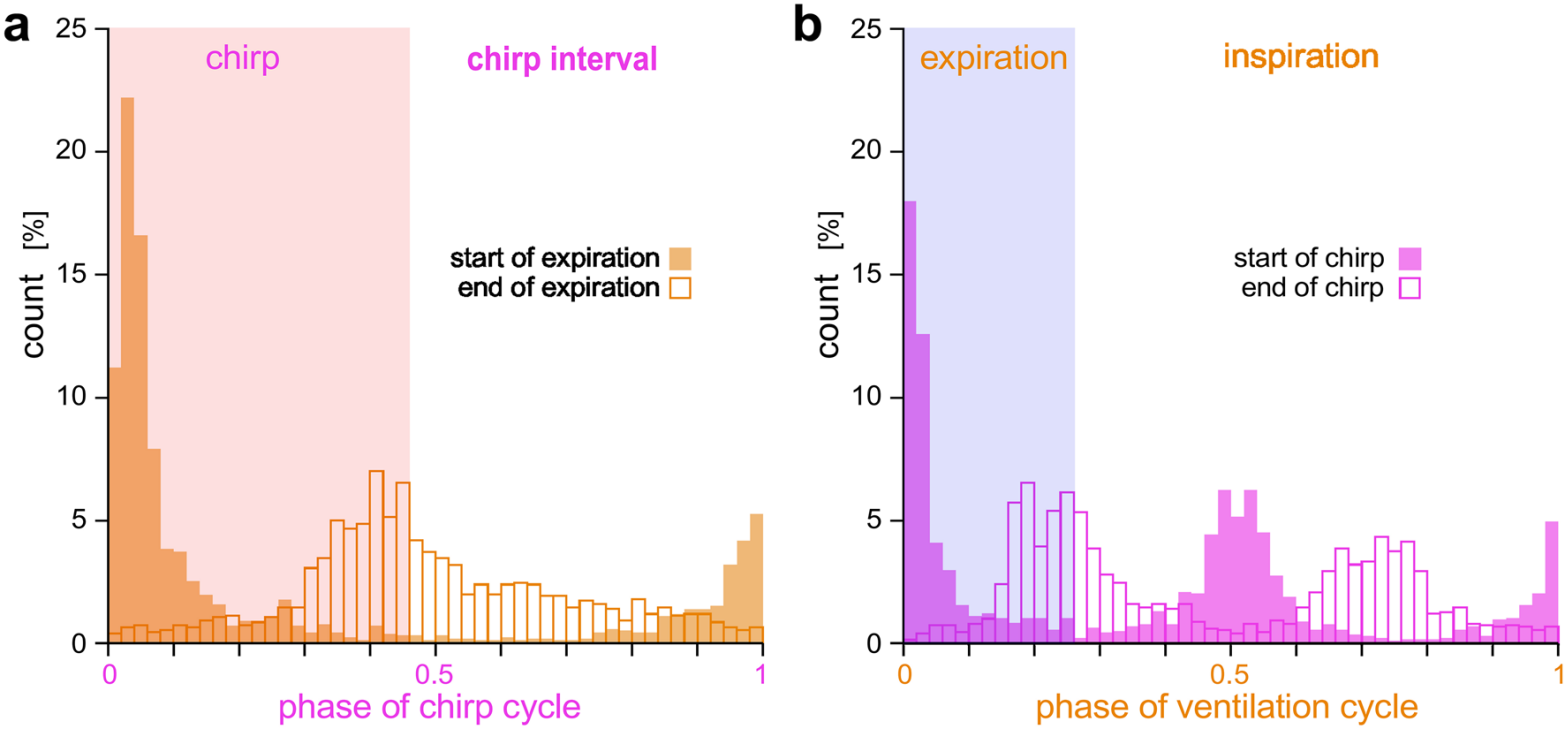
Phase diagrams indicate independence of motor pattern generation for singing and ventilation. **a** Chirp-phase histogram (*n* = 1500 chirp cycles, data set from 10 animals) demonstrates that expiration bursts can occur at any phase within the chirp cycle. However, they are most likely to start with the beginning of a chirp and to cease before the end of a chirp. **b** Ventilation-phase histogram shows that chirps can be generated at any phase within the cycle. They are, however, most likely to begin with the start of the expiration burst when the coupling between chirps and expiration is 1:1 (*n* = 500 ventilatory cycles, data set from 10 animals) or at the start and in the middle of the ventilatory cycle when the coupling pattern is 2:1 (*n* = 500 ventilatory cycles, data set from 10 animals)

A detailed analysis of the timing of both motor patterns revealed the statistics of their phase coupling. The occurrence of the start and end of the expiration motor burst in the phase of the chirp cycle shows that ventilatory bursts can occur at any time within the chirp cycle (Fig. 5a). They are however, most likely to start with the beginning of the chirp (phase 0.9–0.1) and tend to end slightly before the end of the chirp or within the chirp interval, i.e., phase 0.3–0.6 (*n* = 1500 chirp cycles from 10 animals). The distribution of the chirps in the ventilatory cycle shows that although chirps are generated at any phase within the ventilatory cycle, they are most likely to begin with the start of the ventilatory burst when the coupling is strictly 1:1 or at the start and in the middle of the ventilatory cycle (phase 0.5) when the coupling pattern is 2:1 (*n* = 500 ventilatory cycles from 10 animals) (Fig. 5b). In summary, the data presented in Figs. 4 and 5 demonstrate that the chirp pattern and the ventilation pattern are not absolutely coupled, but that in fictive singing cricket preparation both types of CPG motor activities can be generated independently.

### Activity of a singing-CPG interneuron

**Fig. 6:**
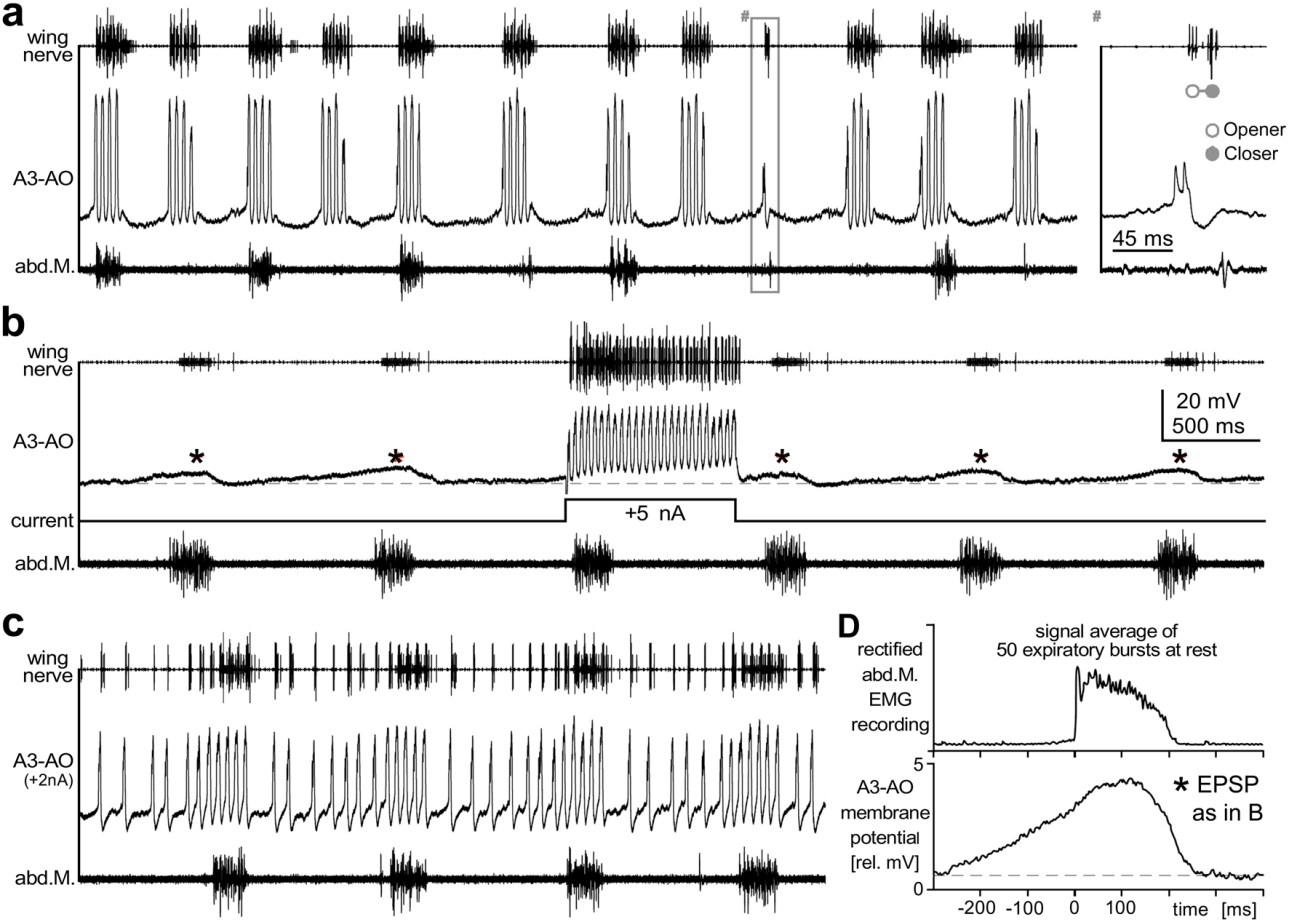
Singing-CPG interneuron receives feedforward excitation coupled to expiration. **a**–**c** Wing nerve recording (upper trace), intracellular recording of the singing-CPG neuron A3-AO (middle trace) and EMG recording of expiratory muscle (bottom trace). **a** During fictive singing, A3-AO depolarized and spiked in phase with the wing-opener motoneurons and hyperpolarized in phase with wing-closer motoneurons. Inset^#^: A3-AO spiking elicits wing-opener and wing-closer activity for one syllable. **b** In a resting cricket, A3-AO receives sub-threshold depolarizing synaptic input with each abdominal ventilation cycle (asterisks; dashed line indicates the resting potential). Intracellular current injection of 5 nA for 850 ms elicits membrane oscillation at about 30 Hz causing rhythmic spiking of the interneuron, which triggers singing activity with a 25-syllable chirp. **c** Constant current injection of 2 nA elicits continuous de- and hyperpolarization cycles with spike bursts during the depolarization. The frequency of the membrane potential oscillations increased significantly during the expiratory phase. **d** Signal average reveals that the rhythmic subthreshold depolarization of A3-AO in the resting cricket (bottom, see * in **b**) starts during the inspiratory interval and reaches its maximum during the expiratory burst (top) before it swiftly decays. Interestingly, the time course of this depolarization in A3-AO closely resembles the spiking pattern of the ventilation-CPG interneuron shown in Fig. 7e

To investigate whether the ventilatory rhythm directly affects the generation of the singing pattern we performed intracellular recordings of the identified A3-ascending opener interneuron (A3-AO) in the abdominal ganglion A3. This interneuron is a crucial element of the pattern generating network for calling song (Schöneich and Hedwig 2012). The membrane potential of A3-AO oscillates with the syllable pattern during fictive singing; it is depolarized and spikes about 10 ms before the wing-opener burst in the wing nerve and it is subsequently hyperpolarized with the wing-closer burst (Fig. 6). Each spike burst in A3-AO leads to the generation of an opener–closer cycle, as exemplified by the generation of an abortive chirp with one syllable (Fig. 6a, inset right), and is followed by a pronounced hyperpolarization. In a resting cricket, intracellular current injection of 5 nA for 850 ms elicited membrane potential oscillation at about 30 Hz coupled with bursts of spikes, and triggered the generation of a motor sequence with 25 syllables (Fig. 6b) (cf. Schöneich and Hedwig 2012).

**Fig. 7:**
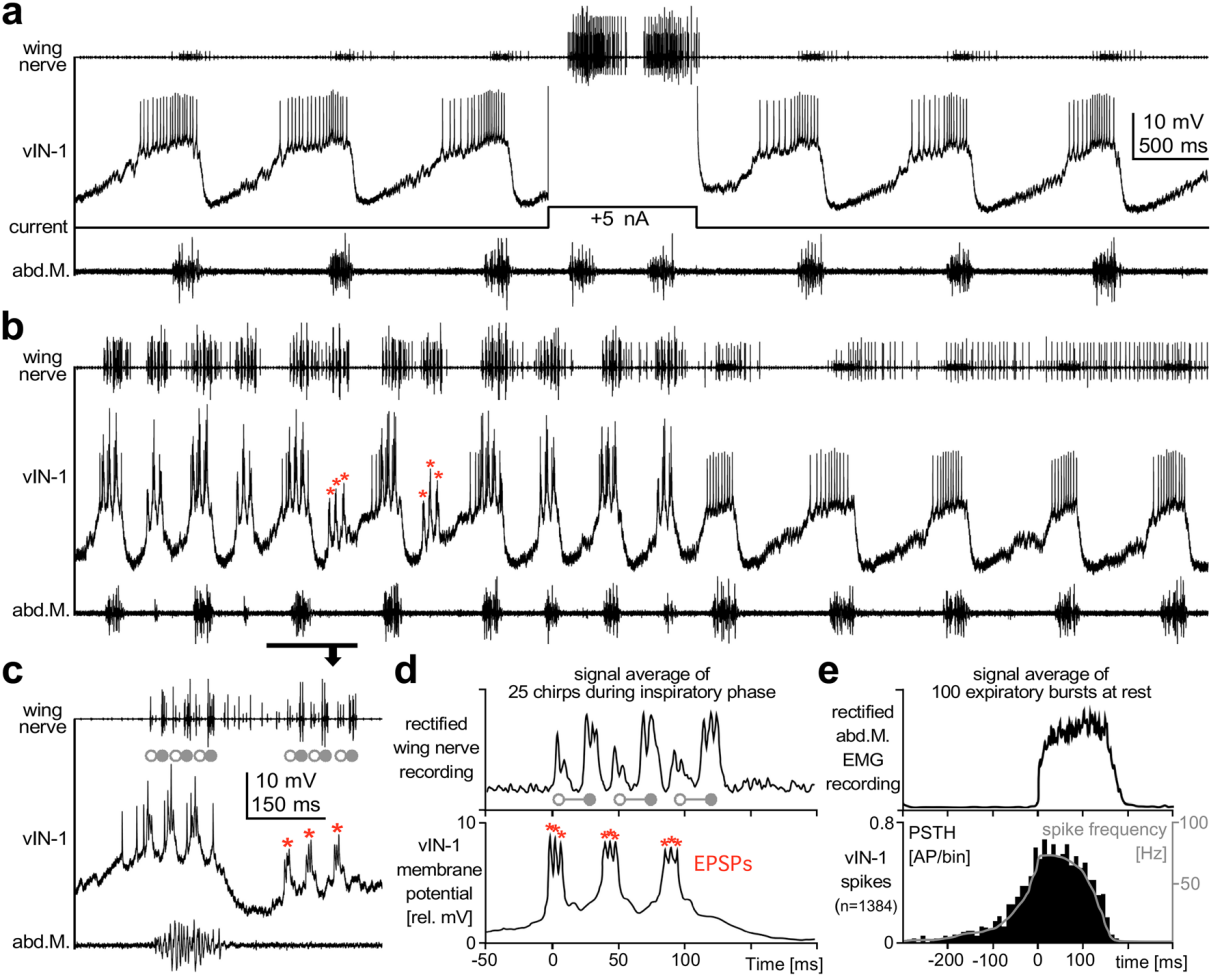
Ventilation-CPG interneuron vIN-1 receives feedforward excitation coupled to fictive singing. **a**–**c** Wing nerve recording (upper trace), intracellular recording of the vIN-1 ventilation-CPG interneuron (middle trace) and EMG recording of expiratory muscle (bottom trace). **a** The ventilation interneuron depolarizes progressively during inspiration and reaches its spiking threshold about 200 ms before expiration. Depolarizing current injection of 5 nA increased the frequency of expiratory bursts by shortening the inspiration intervals. **b**–**d** During fictive singing the vIN-1 neuron receives rhythmic depolarization in the syllable rhythm of the singing pattern. Asterisks mark sub-threshold EPSPs when chirps occur early in the inspiratory interval. **d** Average of 25 chirps occurring during inspiration reveals three EPSP peaks in the opener phase. Wing-opener and wing-closer phase are marked by open and solid circles, respectively. **e** Histogram of vIN-1 spikes for 100 expiration bursts. vIN-1 spiking starts in the inspiration interval and expiratory activity starts when the spike frequency of vIN-1 peaks at 60–70 Hz. Note that the spiking pattern of vIN-1 closely resembles the time course of the feedforward excitation in the singing-CPG interneuron, shown in Fig. 6d

During rest A3-AO received ramp-like subthreshold depolarizing synaptic input tightly coupled to the motor bursts of the ventilation cycle. Constant current injection of 2 nA repeatedly elicited short cycles of de- and hyperpolarization with pronounced spike bursts coupled to the ventilatory activity (Fig. 6c). The frequency of these spike bursts increased significantly from 12 Hz during the inspiratory phase to about 20 Hz during the expiratory phase when the excitatory ventilatory input was strongest (Fig. 6c). Signal averaging with reference to the start of the ventilatory motor burst (Fig. 6c) revealed that the subthreshold depolarization of A3-AO in the resting cricket starts during the inspiratory interval about 250 ms before the generation of the expiratory motor burst. It slowly builds up to reach the depolarization maximum during the muscle activity for abdominal contraction and then decays quickly. Interestingly, this time course of the subthreshold A3-AO depolarization closely resembles the spiking rate of the vIN-1 ventilation-CPG interneuron that we also recorded in A3 (Fig. 7e).

### Activity of ventilation neurons

While probing the A3 ganglion we obtained intracellular recordings from two neurons with activity coupled to the ventilatory rhythm. Each neuron was recorded once. Their morphology was not revealed, but their activity patterns indicate two different functional types.

The ventilatory neuron vIN-1 showed a slowly oscillating membrane potential with a ramp depolarization during the inspiratory interval. Half way through the ramp depolarization, about 200 ms before the expiratory burst, it started to spike and reached a maximum spike frequency of about 75 Hz with the start of the expiratory muscle activity burst (Fig. 7a, e). At the end of the burst, the membrane potential rapidly decayed to the starting level of the ramp depolarization. The activity of this neuron was closely coupled to the generation of the ventilatory cycle. Intracellular depolarizing current injection (5 nA) accelerated the ventilatory cycle by transiently shortening the inspiratory interval (Fig. 7a) and also had an effect on the activity recorded from the wing nerve. As reported in Fig. 2a, the expiratory activity was mirrored in the wing nerve by groups of small amplitude spikes. During the depolarizing current injection into the neuron the motor activity in the wing nerve strongly increased, and motor units with larger spike amplitude were recruited, which resembled the units active during singing.

During fictive singing this ventilation neuron received a rhythmic depolarization coupled to the syllable pattern. A transition from fictive singing to just ventilatory motor activity is shown in Fig. 7b. The wing nerve recording reveals singing motor activity coupled in a 2:1 fashion to the activity of the ventilatory muscle. Every other chirp is accompanied by a very weak or no ventilatory muscle activity. At times of weak ventilatory activity, the neuron’s activity pattern still reflects the chirp pattern, and even when no expiratory muscle activity is generated in phase with a chirp, the neuron receives sub-threshold EPSPs coupled to the syllable pattern (asterisks in Fig. 7b, c).

Averaging the neuron’s membrane potential over 25 chirps occurring during the inspiratory intervals revealed three EPSP peaks in line with the wing-opener phase of each fictive syllable, which indicates an excitatory input from the singing-CPG to this ventilatory-CPG neuron (Fig. 7d). A quantitative analysis of the neuron’s spike activity in reference to the abdominal muscle bursts shows that spiking gradually starts in the inspiratory interval and peaks at about 60–70 Hz at the beginning of the muscle activity burst. Note, the spike rate pattern of the ventilatory-CPG interneuron vIN-1 closely resembles the time course of the ramp-like subthreshold excitation in the singing-CPG interneuron A3-AO, shown in Fig. 6d.

**Fig. 8:**
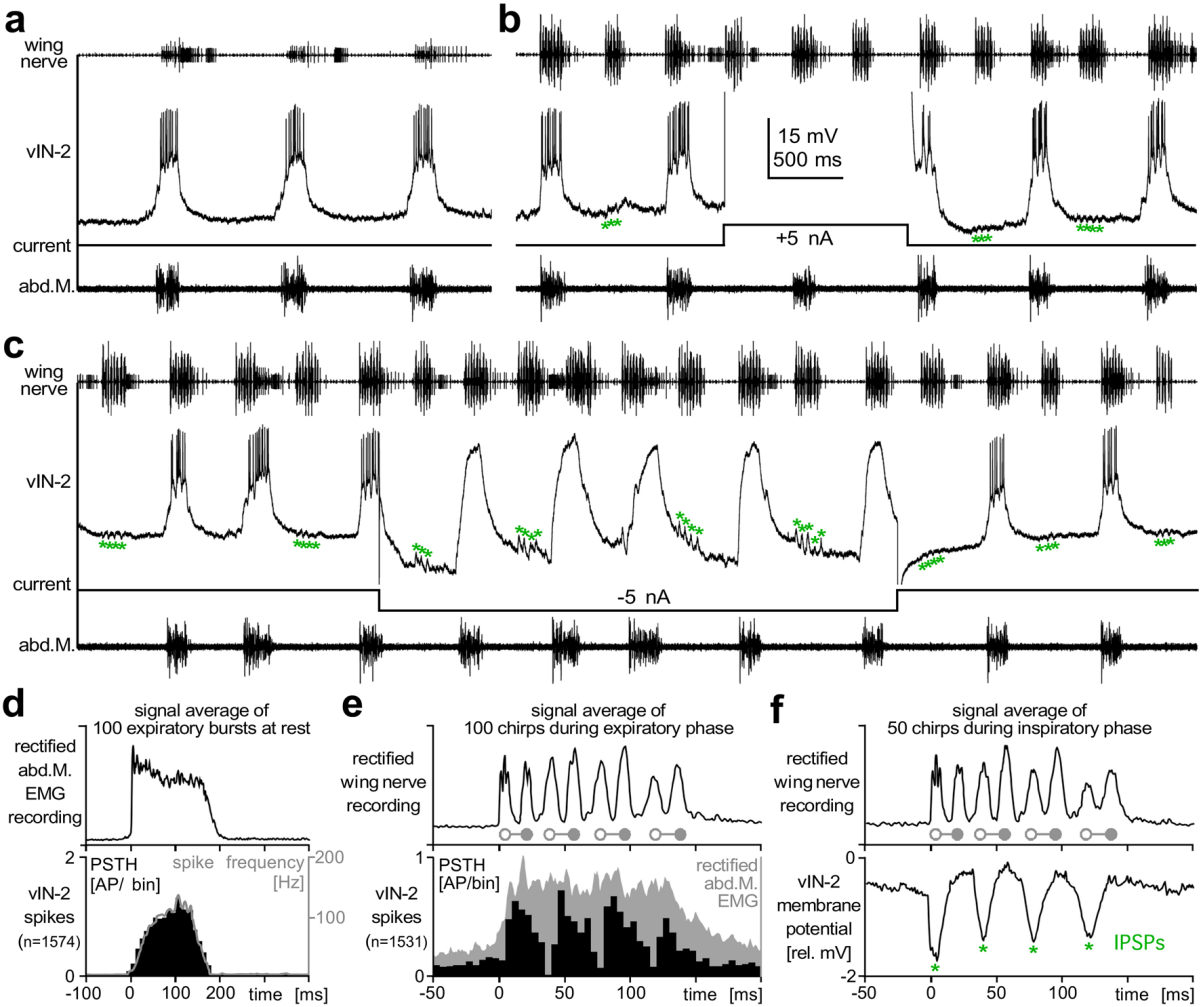
Ventilation interneuron vIN-2 receives a feedforward inhibition coupled to fictive singing. **a**–**c** Wing nerve recording (upper trace), expiration muscle activity (bottom trace) and intracellular recording of the ventilation interneuron vIN-2 (middle trace) before (**a**) and during fictive singing (**b**–**c**). Neither depolarizing (**b**) nor hyperpolarizing (**c**) current injection had an impact on the ventilation or singing activity. **c** Asterisks mark IPSPs in the vIN-2 recording coupled to the syllable pattern, IPSPs are reversed during hyperpolarizing current injection. **d** Increase in spike frequency of vIN-2 does not precede expiration and peaks in the second half of the burst. Signal average for 100 expiration bursts during rest. **e**–**f** vIN-2 receives feedforward inhibition in the opener-phase that rhythmically interrupts its spiking activity during expiration. Wing-opener and wing-closer activity are marked by open and solid symbols, respectively. **e** Signal average of vIN-2 spike activity for 100 chirps which occurred during expiration. **f** Signal average of vIN-2 membrane potential for 50 chirps which occurred during inspiration intervals

The ventilatory neuron vIN-2 was activated in phase with expiration and started spiking with the onset of the expiratory burst. It reached a maximum spike rate of about 120 Hz half way through the EMG burst, and its activity then declined with the end of expiration (Fig. 8a, d). We manipulated the neuron activity during fictive singing by depolarizing and hyperpolarizing current injection of 5 nA. Neither of the current pulses caused a significant effect on the ongoing singing or ventilatory activity (Fig. 8b, c). We therefore rule out that this was a ventilatory-CPG interneuron.

While the neuron was recorded, a 2:1 coupling between the chirp and the expiration activity occurred. During those chirps, which were not represented in the ventilatory motor activity, the neuron received an inhibition in phase of the syllable pattern, IPSPs reversed in amplitude when the neuron was hyperpolarized (Fig. 8c, asterisks). This inhibitory input during the inspiration phase was analyzed by averaging the membrane potential in reference to the chirps (Fig. 8f), demonstrating that IPSPs occurred in phase with the wing-opener activity of each syllable within the fictive chirps. When the chirp was coupled to ventilation, the neuron showed a salient depolarization and although its spike activity appeared as in the resting animal, the activity was modulated by an inhibitory input. Analyzing the distribution of spike activity in reference to the chirps revealed that spiking was drastically reduced during the wing opener phase of each syllable of the fictive chirps, while the expiratory muscle activity was not modulated (Fig. 8e).

## Discussion

The coupling of CPGs controlling different motor patterns poses a fundamental question in systems neurobiology (von Holst 1943; Dickinson 1995; Clarac and Pearlstein 2007). Here we used fictively singing crickets to explore the link between the singing-CPG and the CPG for abdominal ventilation. Our wing nerve and EMG recordings in conjunction with the synaptic interneuronal activity allow insight into the underlying neural coupling mechanism.

Although each type of ventilatory interneuron was only intracellularly recorded once, the quality of these recordings and the effects of intracellular current injection allow a clear functional insight into the coupling mechanisms between the singing- and ventilatory-CPG. We acknowledge that future studies are required to reveal the structure of the ventilatory interneurons and to corroborate our findings; we think, however, that the details of synaptic and spike activity in our recordings provide strong evidence for a feedforward mechanism of motor patter coordination in singing crickets.

### Evidence from EMG and nerve recordings

In resting crickets, ventilation is driven by abdominal expiratory pumping movements, while during singing (*G. bimaculatus, G. campestris*) the cycle period of ventilation shortens and expiration becomes tightly coupled to the generation of chirps (Huber 1960; Kutsch 1969). Some vertebrates experience mechanical benefits by coupling locomotor activity and breathing (Berger et al. 1970; Bramble and Carrier 1983; Nassar et al. 2001) and for singing, coupling with breathing is even a necessity (Andalman et al. 2011). A detailed discussion of the functional background for coupling sound production and ventilation in crickets, is given by Paripovic et al. (1996), it may be related to a higher metabolic rate, which in singing crickets significantly increases (Prestwich and O’Sullivan 2005; Mowles 2014).

Our EMG recordings demonstrate that in resting and in singing *G. bimaculatus* changes in the ventilatory period are based on increasing the duration of the inspiratory interval, while the duration of the expiratory bursts remains constant (Fig. 2). Such a relationship also occurs in *G. campestris* and in *Teleogryllus commodus* (Paripovic et al. 1996) and in locusts (Lewis et al. 1973; Burrows 1996) and seems to be characteristic feature of the ventilatory network. Also the increase in the chirp period is driven by an increase in the interchirp interval, as the duration of chirps composed of the same syllable number shows only a minor increase with increasing chirp period (Fig. 3). Thus, both motor patterns share a common characteristic: increases in cycle period are linked to an increase in the “silent” intervals between the bursts of motor activity, which in both cases may be linked to a gradually increasing ramp-like depolarizations that leads to the generation of the motor activity (Otto and Janiszewski 1989; Ramirez and Pearson 1989a; Jacob and Hedwig 2019).

Our and previous EMG recordings (Kutsch 1969; Paripovic et al. 1996) show that chirps and expiratory bursts are usually phase coupled in a strict 1:1 or 2:1 pattern, with expiration starting at the beginning of a chirp and terminating with its end, and chirps starting with the expiration or half way through the ventilatory cycle, when a 2:1 coupling occurs (Figs. 4, 5). The coupling of the motor output is not shifting like in relative coordination, it is rather restricted to specific phases of the motor cycles, even at times during very high or low chirp rates, when both patterns can occur in antiphase (Fig. 4), indicating that the motor patterns settle with different preferred states of absolute coordination (von Holst 1943). Due to the tight coupling of the patterns in naturally singing males, Kutsch (1969) proposed that ventilation and singing are both driven by a common slow oscillatory network. However, EMG recordings in fictive singing crickets at least occasionally show a wider range of coordination states, which demonstrate that both patterns cannot be driven by the same neuronal network. An extended range of coordination during fictive singing may be supported by a lack of sensory feedback, occurring during normal ventilation (Hustert 1975), but also Paripovic et al. (1996) looking at intact crickets mention exceptions from the standard coupling of the motor patterns. Moreover, the hypothesis of shared basic rhythm generators for flight and singing (Kutsch 1969) contrasts with the finding that the same forewing motoneurons are driven by separate pools of premotor interneurons for flight and singing as interneurons of the flight motor network are inhibited during singing and vice versa (Hennig 1990).

### Evidence from intracellular recordings

Furthermore, our intracellular recordings also indicate that both patterns are generated by independent CPGs, which at the level of the abdominal ganglia reciprocally share excitatory drive. The synaptic activity demonstrates that CPG interneurons of both networks are coupled by mutual feedforward excitation. In resting crickets, the singing-CPG interneuron A3-AO receives a ramp-like increasing excitatory synaptic input in phase with the ventilatory cycle, which peaks with the expiration burst (Fig. 6b, d). A slight constant depolarization of the interneuron by current injection, enhances the effect of the ventilatory input to the singing-CPG interneuron and leads to bursts of motor activity in the wing nerve, akin to the motor activity during singing. Thus, the ventilatory-CPG will enhance the probability for generating a chirp in phase with the expiration activity. As the spike rate of the ventilatory interneuron vIN-1 (Fig. 7e) matches the profile of the slow ventilatory depolarization in the singing-CPG interneuron (Fig. 6d), it may also provide this excitatory input. Based on this link, one might expect that in normal singing crickets the timing of chirps, especially at the beginning of a singing bout when the singing-CPG is not yet fully activated, is determined by the expiration pattern.

The synaptic activity forwarded from the singing-CPG to the ventilation-CPG interneuron is coupled to the fictive chirps and the pattern of fictive syllables. The vIN-1 ventilation-CPG interneuron received excitatory synaptic inputs, while the vIN-2 ventilation neuron, which is not part of the ventilation-CPG, received inhibition in the opener phases of the syllable pattern. The timing of vIN-2 inhibition is very similar to the corollary discharge inhibition in cercal giant interneurons during singing (Schöneich and Hedwig 2015). However, both synaptic connections may contribute to structure the expiratory bursts in the pattern of the fictive syllables. A modulation of the expiratory muscle activity in the syllable pattern of the chirps also occurs in *G. campestris* (Otto and Weber 1982; Paripovic et al. 1996) and may fine-tune the ventilatory motor output to the biomechanical demands of the singing cricket.

### Functional implications

Although both motor patterns may occasionally occur in a very unstable coupling (Fig. 4c) our data indicate that driven by the faster chirp pattern the input to the slow ventilatory ramp depolarization in the ventilation-CPG interneuron leads to the premature generation of expiratory bursts, and an increase of the ventilation rhythm, stabilizing both CPGs with a 1:1 coupling. If premature chirps are generated the feedforward excitation to the ventilation-CPG will be lower, and expiration bursts coupled to the singing pattern will fail. However, driven by the ventilation-CPG the next ventilatory burst is generated with a stronger amplitude and due to the feedforward excitation to the singing-CPG the subsequent chirp is generated with a full amplitude coupled to the ventilation cycle. In this way a 2:1 coupling between chirps and expiration may be established. Anti-phase coordination between singing and ventilation are a rare exception and only occurred at very low chirp rates. In this case, the ramp-like depolarizations in both motor networks may develop very slowly, and if the depolarization is too low the mutual feedforward may not effectively entrain both motor network. These states of coordination may also be subject to descending commands, as ventilation and singing are controlled by interneurons from the cephalic ganglia (Otto and Weber 1982; Otto and Janiszewski 1989; Hedwig 2000).

Ventilation and singing are also coupled at the level of the suboesophageal ganglion. Identified descending interneurons are inhibited in phase with expiration and in singing crickets also in phase with the generation of syllables; while depolarizing the interneurons reduces ventilation and singing activity. These neurons apparently receive inhibitory feedback from both CPGs, they might contribute to coordinating both motor patterns, but more likely are involved in controlling motor activity in general (Otto and Weber 1982; Otto and Hennig 1993).

The synaptic activity underlying the coordination between both CPG networks reveal centrally generated feedforward commands, which couple and structure the output of both motor networks. Feedforward mechanisms are widespread in networks (Milo et al. 2002) and can be found at the circuit and cellular level in nervous systems (Houk 1988; Abbott and Regehr 2004; Schafer 2016). Corollary discharges from one motor system can shape the activity in other motor networks (Parker 2003; Hänzi et al. 2015; Straka et al. 2018) or as an efference copy prepare sensory pathways to the consequences of self-generated activity (Poulet and Hedwig 2007; Schöneich and Hedwig 2015; Chagnaud et al. 2015). In the insect CNS, the ventilatory pattern widely “irradiates” into the activity of other motor networks and vice versa (Miller 1966; Burrows 1975b; Ramirez 1998). In crickets and in locusts thoracic motoneurons receive synaptic input coupled to the ventilatory rhythm and a simultaneous representation of the ventilatory and the faster flight motor pattern occurs (Bentley 1969). During flight, this ventilatory synaptic activity—besides the input from the flight CPG—may contribute to shape the activity patterns of the motor neurons (Burrows 1975a, b). With the onset of flight, the rate of abdominal ventilation increases and a feedforward mechanism from the flight initiating interneurons is suggested to prepare the ventilatory system for the change in motor activity (Ramirez and Pearson 1989b). Also during flight the activity pattern of a prothoracic auxiliary ventilation motoneuron and of descending ventilation interneurons of the SOG changes, and they strictly discharge in phase with the flight motor rhythm. This is regarded as a reconfiguration of the ventilatory network (Ramirez 1998), a functional change which goes beyond the feedforward coupling occurring in singing cricket.

### Evolution of ventilation and singing

The generation of the ventilatory motor pattern in crickets and in locusts is distributed along the CNS (Huber 1960; Lewis et al. 1973; Ramirez and Pearson 1989a; Burrows 1996), and even isolated abdominal ganglia can generate the basic ventilatory motor output (Miller 1966; Lewis et al. 1973; Hustert and Mashaly 2013). While the motoneurons recruited during singing are housed in the mesothoracic ganglion (Elepfandt 1980; Hennig 1990) the network of the singing-CPG in crickets is organized along the abdominal nerve cord, with different ganglia contributing specifically towards the generation of the chirp and syllable pattern (Schöneich and Hedwig 2011, 2012; Jacob and Hedwig 2016). The organization of the ventilation network and the singing-CPG along the chain of abdominal ganglia together with their feedforward coupling may allow speculations about a possible evolutionary link. Evolutionary considerations point towards continuous pulse patterns (i.e., trills) as the archetype of cricket song, from which more complex temporal patterns evolved (Alexander 1962). An earlier suggestion was that singing may have evolved from flight behavior (Huber 1962), has not been supported by neuronal evidence (Hennig 1990; Schöneich and Hedwig 2012). Due to the synaptic coupling between the ventilation and the singing network, we speculate that the evolution of chirp patterns may have been supported by feedforward activity from the ventilation network to the singing network, enhancing the probability for the generation of sound pulses, whenever an expiratory burst was to be generated. The evolution from the ancient songs with continuous pulse patterns to more complex chirp patterns might have occurred by integrating the ventilatory feedforward into the singing network.
