# Correction to: Feedforward discharges couple the singing central pattern generator and ventilation central pattern generator in the cricket abdominal central nervous system

**DOI:** 10.1007/s00359-019-01388-4

**Published:** 2020-01-13

**Authors:** Stefan Schöneich, Berthold Hedwig

**Affiliations:** 1 Department of Zoology, University of Cambridge, Cambridge, UK; 2 Institute of Zoology and Evolutionary Research, Friedrich-Schiller-University Jena, Jena, Germany

## Correction to: Journal of Comparative Physiology A (2019) 205:881–895 10.1007/s00359-019-01377-7

The authors listed the following reference:

Burrows M (1996) Breathing. In: Burrows M (ed) The neurobiology of an insect brain, chap 12. Oxford University Press, Oxford, pp 563–596

M. Burrows is the sole author of the book, an editor was not involved. The reference should read:

Burrows M (1996) Chapter 12: Breathing. In: The neurobiology of an insect brain. Oxford University Press, Oxford, pp 563-596

The authors apologise for citing incorrectly.

